# A parametric investigation on traditional and cortical bone trajectory screws for transpedicular fixation

**DOI:** 10.1186/s12891-022-05477-5

**Published:** 2022-06-27

**Authors:** Tzu-Tsao Chung, Chen-Lun Chu, Dueng-Yuan Hueng, Shang-Chih Lin

**Affiliations:** 1 Graduate Institute of Applied Science and Technology, National Taiwan University of Science and Technology, Keelung Rd, No. 43, Sec. 4, Taipei, 106 Taiwan, Republic of China; 2 Department of Surgery, Cheng-Hsin General Hospital, Taipei, Taiwan; 3 Department of Neurological Surgery, Tri-Service General Hospital, National Defense Medical Center, Taipei, Taiwan; 4 Department of Orthopaedic Surgery, Shin Kong Wu Ho Su Memorial Hospital, Taipei, Taiwan; 5 Graduate Institute of Biomedical Engineering, National Taiwan University of Science and Technology, Taipei, Taiwan

**Keywords:** Cortical bone trajectory, Adjacent segmental disease, Pedicle screw, Transpedicular fixation, Finite element analysis

## Abstract

**Background:**

Many studies have been conducted to compare traditional trajectory (TT) and cortical bone trajectory (CBT) screws; however, how screw parameters affect the biomechanical properties of TT and CBT screws, and so their efficacy remains to be investigated.

**Methods:**

A finite element model was used to simulate screws with different trajectories, diameters, and lengths. Responses for implant and tissues at the adjacent and fixed segments were used as the comparison indices. The contact lengths and spanning areas of the inserted screws were defined and compared across the varieties.

**Results:**

The trajectory and diameter had a greater impact on the responses from the implant and tissues than the length. The CBT has shorter length than the TT; however, the contact length and supporting area of the CBT within the cortical bone were 19.6%. and 14.5% higher than those of the TT, respectively. Overall, the TT and CBT were equally effective at stabilizing the instrumented segment, except for bending and rotation. The CBT experienced less adjacent segment compensations than the TT. With the same diameter and length, the TT was considerably less stressed than the CBT, especially for flexion and extension.

**Conclusions:**

The CBT may provide less stress at adjacent segments compared with the TT. The CBT may provide more stiffer in osteoporotic segments than the TT due to greater contact with cortical bone and a wider supporting base between the paired screws. However, both entry point and insertion trajectory of the CBT should be carefully executed to avoid vertebral breach and ensure a stable cone-screw purchase.

## Introduction

Transpedicular screw fixation has been a common treatment for spinal instability and pathologies such as scoliosis, spondylolisthesis, trauma, neoplasms and other degenerative diseases [1]. In general, two techniques have been used to insert screws into a pedicle: traditional trajectory (TT) and cortical bone trajectory (CBT). The TT screw follows the anatomic axis of the pedicle into the cancellous bone of the vertebral body, whereas the CBT screw follows a laterally directed path to meet the cortical bone within the pedicle and cortical shell at the screw tip [2].

**Fig. 1:**
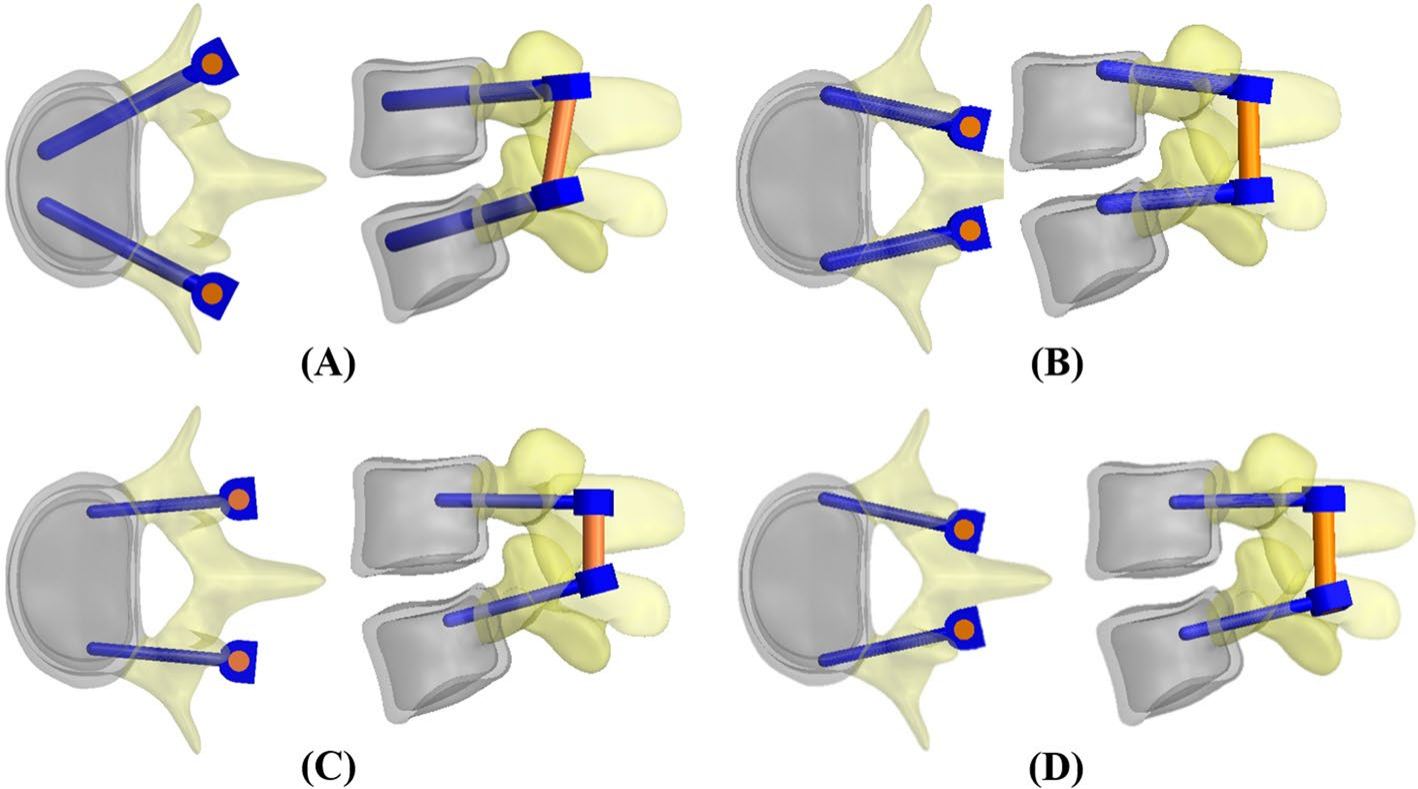
Four varieties of screw trajectory were investigated in this study. **A** TT. **B** CBT. **C **AIS. **D** MLT. Both AIS and MLT trajectories were defined in the content and designed to evaluate the effects of screw trajectory

The TT and CBT show large differences in parameters (trajectory, diameter, and length) within the vertebral body and posterior element (Fig. 1). The paired screws of the TT show convergent trajectories, while the screws of the CBT show divergent trajectories, and have been demonstrated to narrow the surgical wound and reduce deterioration of the multifidus muscle [3]. In specific, the CBT has been reported to result in similar or decreased postoperative pain and blood loss when compared with the TT [4, 5]. When comparing biomechanical properties [6–9], Li et al*.* found that the 4.5 mm CBT screw provided a stronger purchase to osteoporotic vertebrae than the 6.5 mm TT screw and decreased the likelihood of damage to the facet joint [6]. Similarly, using deer vertebrae, Oshino et al. noted that 4.5 mm CBT screws were comparable or even superior in immobilizing ability than the 6.5 mm TT screws [7]. In a systematic review and meta-analysis, the pain scores and fusion ratio were not significantly difference between the two techniques, and CBT was found to be superior in operation time, length of stay and incidence of adjacent segmental disease (ASD) [8]. Similar positive conclusions pertaining to CBT have been reported by other clinical studies [3–5, 9, 10].

Nevertheless, not all reports on CBT have been optimistic or yielded promising findings [11–14]. Even with the stronger purchase to cortical bone in osteoporotic bone density, both entry point and insertion angle need to be prepared well and the CBT screw placement performed cautiously [11]. If screw placement is not done accurately, the aforementioned biomechanical advantages of the CBT system will fail to arise, which is why a 3D-printed surgical guide has been created in order to enhance the precision of the entry point, insertion angle and screw length [12, 15, 16]. With a lateral and upward trajectory, moreover, the CBT is shorter and slimmer than the TT; however, these slimmer screws are capable of reducing holding power, fatigue strength, and immobilizing ability. On account of this, Dayani et al*.* observed that patients with a pedicle diameter of less than 7 mm should avoid using the CBT screw fixation in order to reduce a breach of the lateral vertebral body [13], and Gonchar et al*.* reported that breakage of a 4.75 mm CBT screw could be eliminated if its diameter was increased to 5.5 mm [14]. While interesting and informative, these studies do not address how the parameters of the two systems affect the tissue responses and behaviors of the implants, and thus this served as the prime motivation for our study.

The present study aimed to gain insight into the TT and CBT mechanisms modulating tissue responses and behaviors of the implants. Rather than relying upon a single vertebrae or motion unit, the entire lumbosacral column was adopted to simulate the kinematic and kinetic changes at fixed and adjacent segments. The screw parameters varied systematically, and the contact length and spanning area of the screws within the vertebrae were formulated to help clarify the differences in results between TT and CBT in the literature.

## Methods

### Finite element models from L1 to S1 segments

**Fig. 2:**
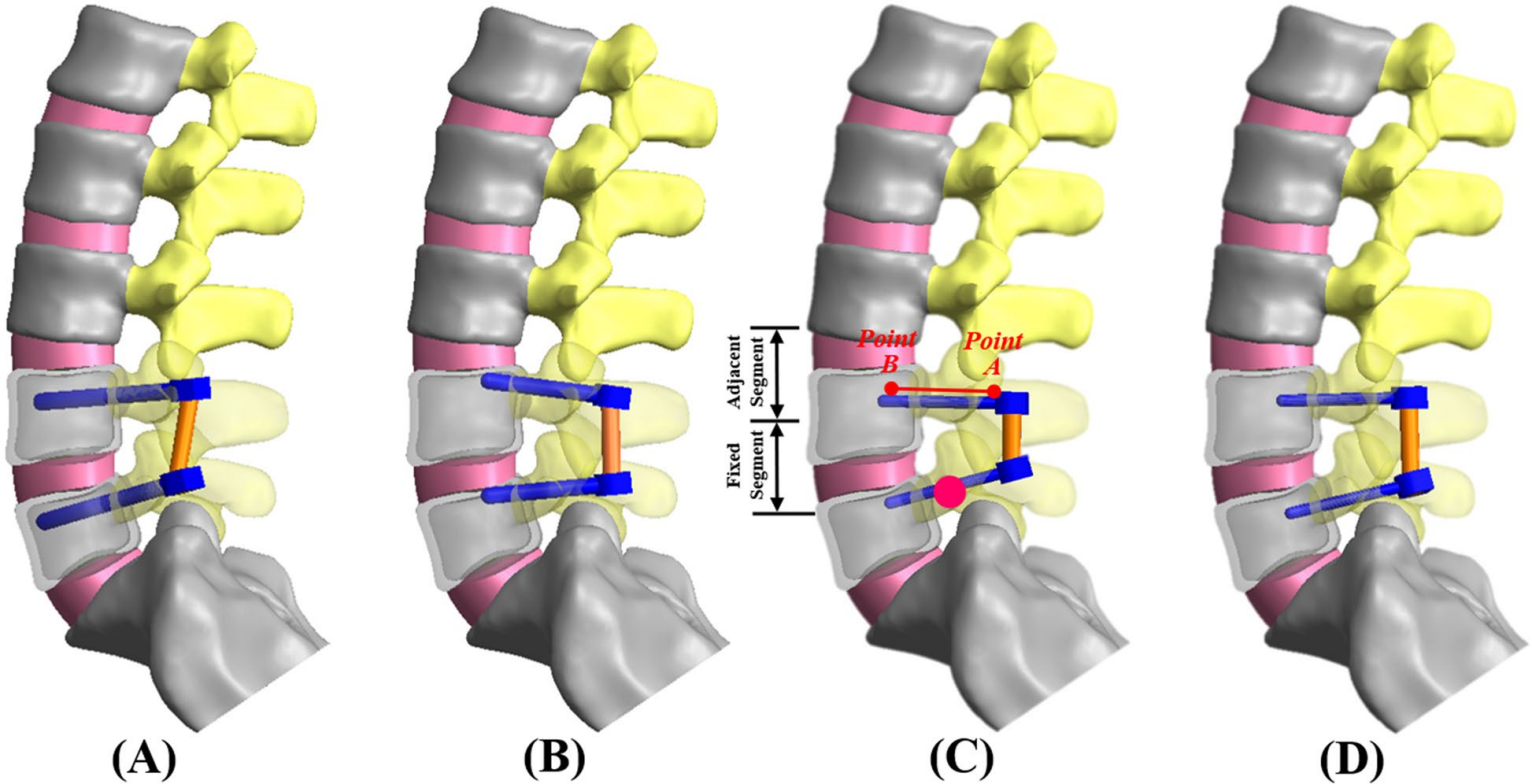
Finite-element models of the four screw trajectories. **A** TT. **B** CBT. **C** AIS. **D** MLT. The definitions of the *Line AB* and the *Points A* and *B* were described in the content

This study used a validated finite element model to evaluate the effects of the screw parameters on lumbar biomechanics [17–19]. A three-dimensional (3D) lumbosacral model was developed based upon the computed tomography (CT) images of healthy segments from L1 to S1 which showed no any degeneration or deformity. The lumbosacral models were instrumented into the transpedicular fixator without an intervertebral fusion device (Fig. 2). Fixation of the transpedicular screw was divided into four trajectories: TT, CBT, Average Insertion Site (AIS) and Mediolateral Trajectory (MLT) (Fig. 1). The TT and CBT represented the commercialized screws (Figs. 1A and B), as this study focused on comparisons between these two screw types in terms of trajectory, diameter and length. For the first parameter, the AIS and MLT were designed specifically to evaluate effects of screw trajectory; AIS indicates the screw insertion point midway between TT and CBT (Fig. 1C), and MLT serves as the control group for CBT not to cross the bilayered bone cortex (Fig. 1D). For the other two parameters, four specifications of diameter and length were adopted: 3.5 and 5.0 mm in diameter, 35 and 50 mm in length. In total, three parameters comprised seven varieties: three TT (TT_3.5–35_, TT_3.5–50_, TT_5.0–50_), two CBT (CBT_3.5–35_, CBT_5.0–35_), one AIS (AIS_3.5–35_) and one MLT (MLT_3.5–35_). All fixators were instrumented into the healthy L4-L5 segment (Fig. 2).

For all varieties, the titanium-based rods were consistently 5.5 mm in diameter to ensure equal comparisons. The top- and side-view trajectories of the TT, CBT, AIS, and MLT are shown in Fig. 1; *Line AB* denotes the stress distribution along the screw shaft (Fig. 2C), *Points A* and *B* were, respectively, located at the screw tip and hub (the junction between the smooth and threaded regions).

Configurations for the implants were developed by SolidWorks, Ed. 2018 (SolidWorks Corporation, Concord, MA, USA). The screw threads were excluded from the simulation since screw slippage was not a major concern of this study. No screw was cannulated. For computational simplification, the set screws were omitted and rods were assumed to be fully embedded in the heads of the pedicle screws. The metal components of the fixators were made from titanium-based alloy (Ti-6Al-4 V ELI). The assumption of linear elasticity was assigned for all implant materials and further validated by comparing the calculated von Mises stresses with the strength of the Ti-6Al-4 V alloy.

### Finite element analyses

The lumbosacral model was fixed at the S1 bottom and loaded at the L1 top to activate flexion, extension, right bending, and rotation. The interfaces of the facet joints were modeled as surface-to-surface contact elements, allowing for separation and slippage. The bone-screw interfaces were modeled as firmly purchasing and the von Mises stresses along *Line AB* were calculated (Fig. 2C). The displacement applied to the L1 vertebra ensured the disc range of motion (ROM) for the adjacent and fixed segments of the healthy model would be comparable to that of cadaveric data gathered by Yamamo et al*.* [20]. Models were meshed using the ten-node tetrahedral solid elements, and mesh refinement was carried out for modeling accuracy until a monotonic convergence with less than a 5% difference in the total strain energy was achieved. A nonlinear algorithm with a large deformation and Direct Sparse solver was used by Simulation Ed. 2018 (SolidWorks Corporation, Concord, MA, USA).

Four indices were chosen to evaluate the trajectory-, diameter- and length-related effects of the TT and CBT screws on tissue responses and behaviors of the implants. Tissue responses at the fixed and adjacent segments were evaluated in terms of disc ROM, disc stress, and facet force. The disc ROM is defined as the difference between disc angles before and after lumbar motion. The stress distributions at the bone-screw interfaces (*Line AB*) were used as the indices for fatigue breakage and loosening failure. The von Mises stress was chosen as the equivalent stress for discs and screws.

## Results

**Fig. 3:**
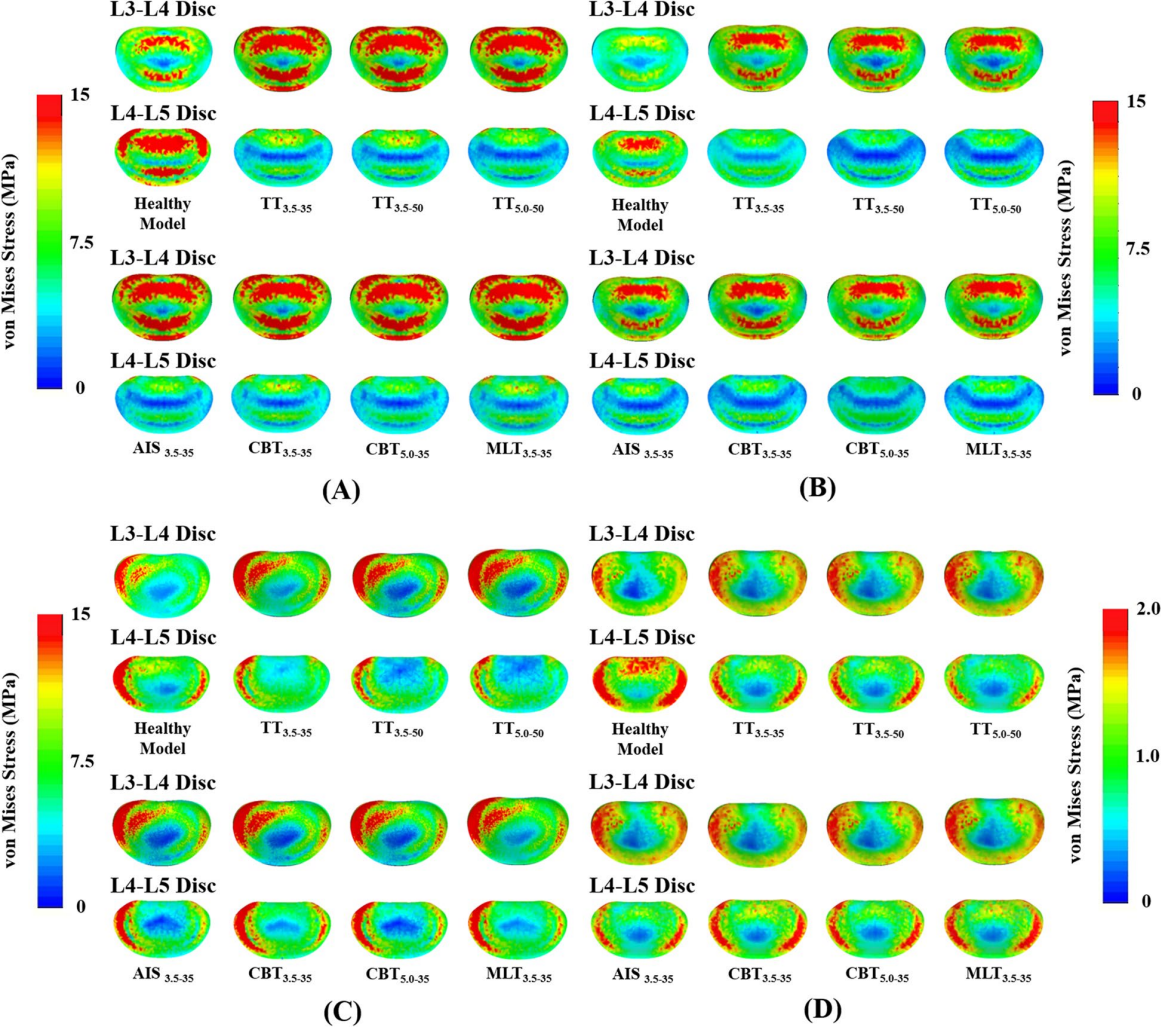
Stress contours at the L3-L4 (upper) and L4-L5 (lower) discs of the different fixators. **A** Flexion. **B** Extension. **C** Bending. **D** Rotation. The upper and lower were the healthy and instrumented models, respectively. The stress scales were different for (**A, B, and C**) and (**D**) for visual clarity

**Fig. 4:**
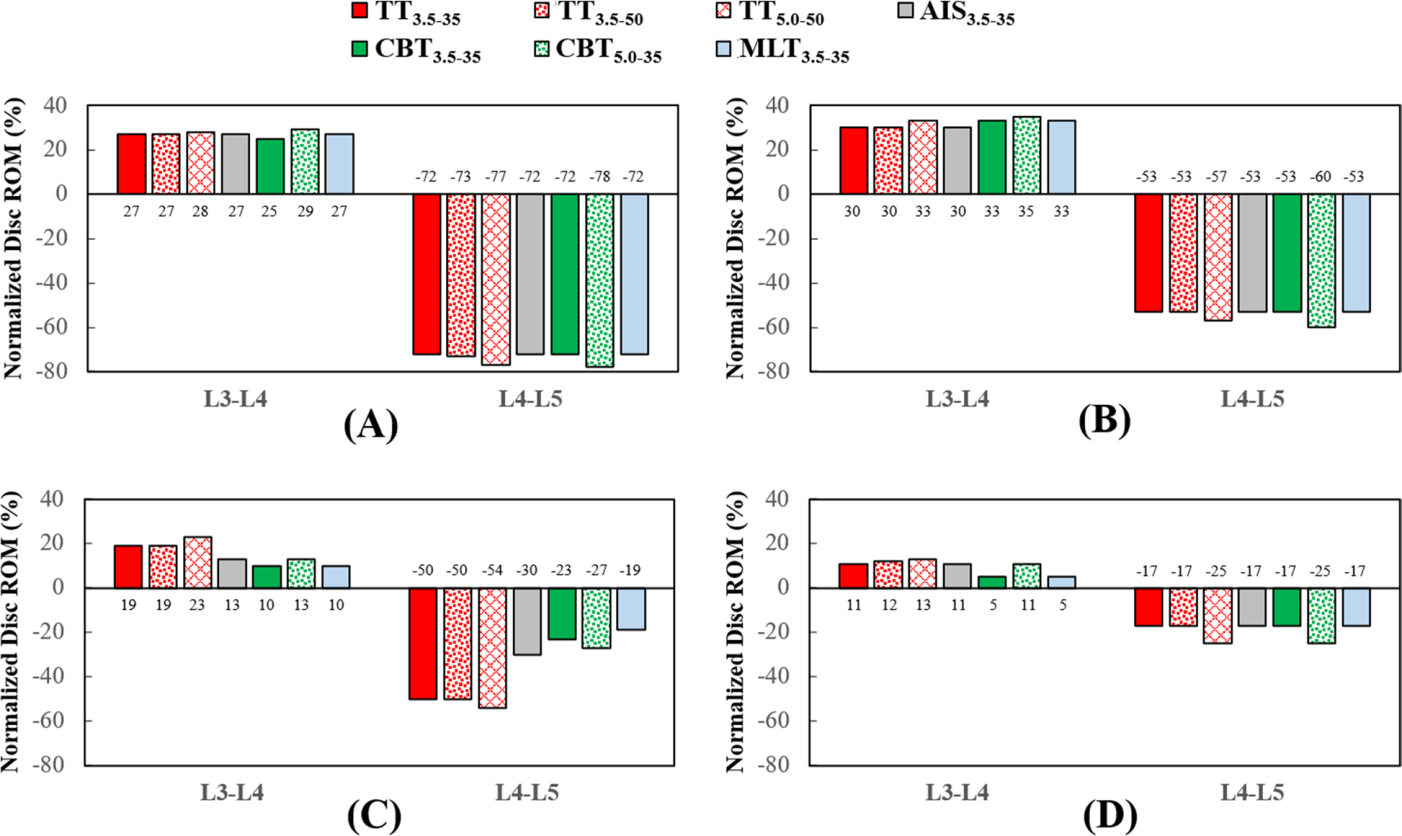
Normalized disc ROMs of the different fixators at the adjacent and fixed segments. **A** Flexion. **B** Extension. **C** Bending. **D** Rotation

**Fig. 5:**
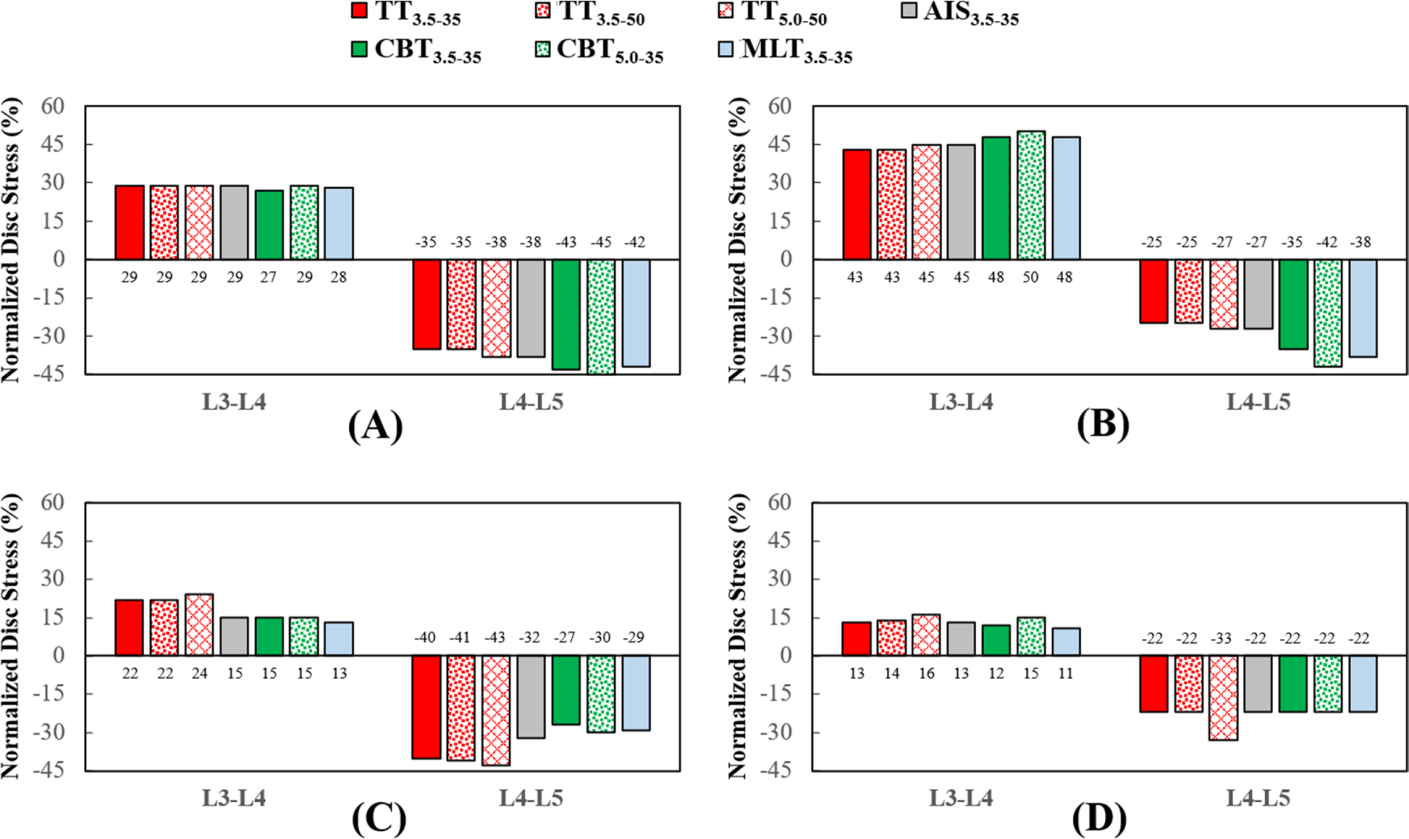
Normalized disc stresses of the different fixators at the adjacent and fixed segments. **A** Flexion. **B** Extension. **C** Bending. **D** Rotation

**Fig. 6:**
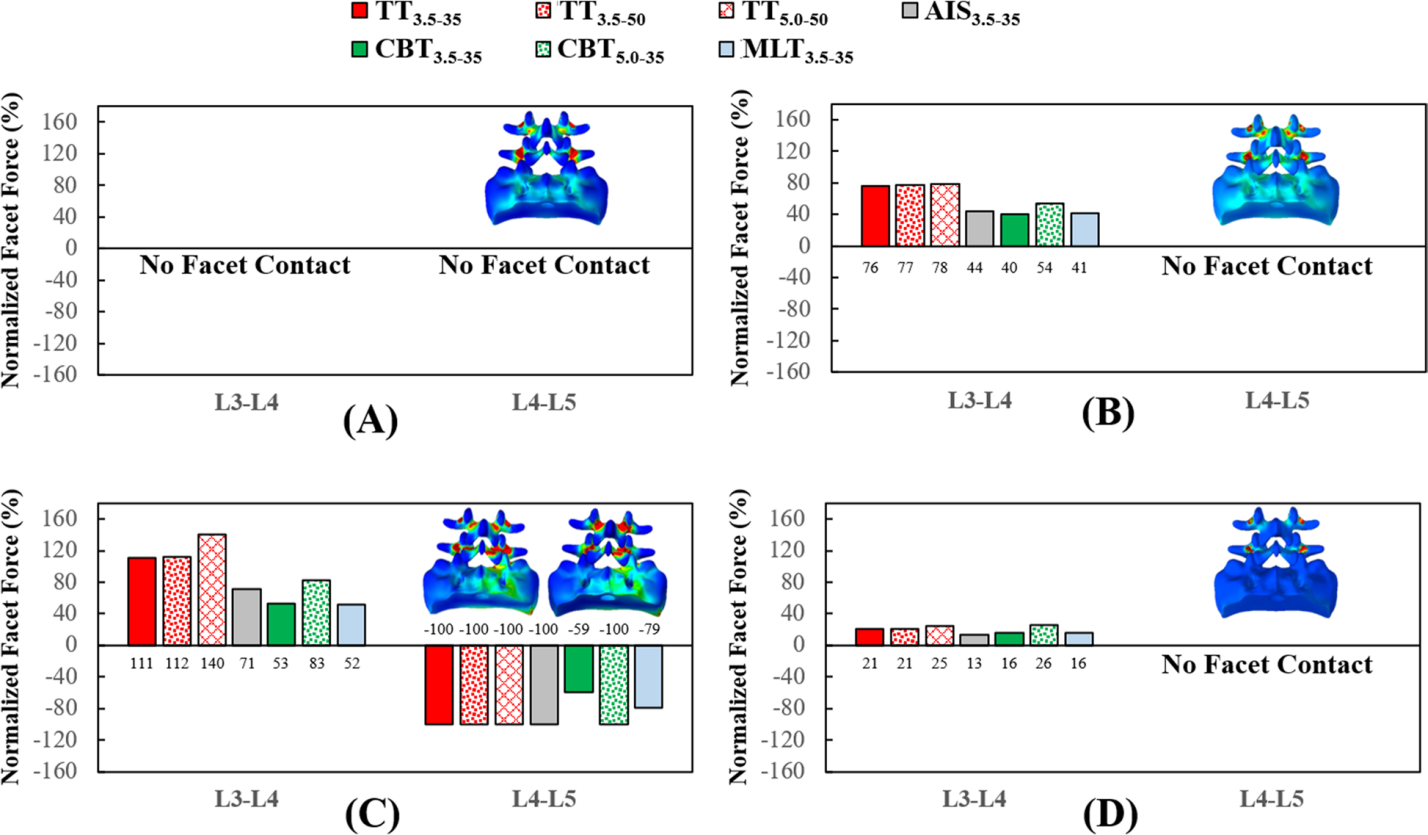
Normalized facet forces of the different fixators at the adjacent and fixed segments (**A**) Flexion. **B** Extension. **C** Bending. **D** Rotation

**Fig. 7:**
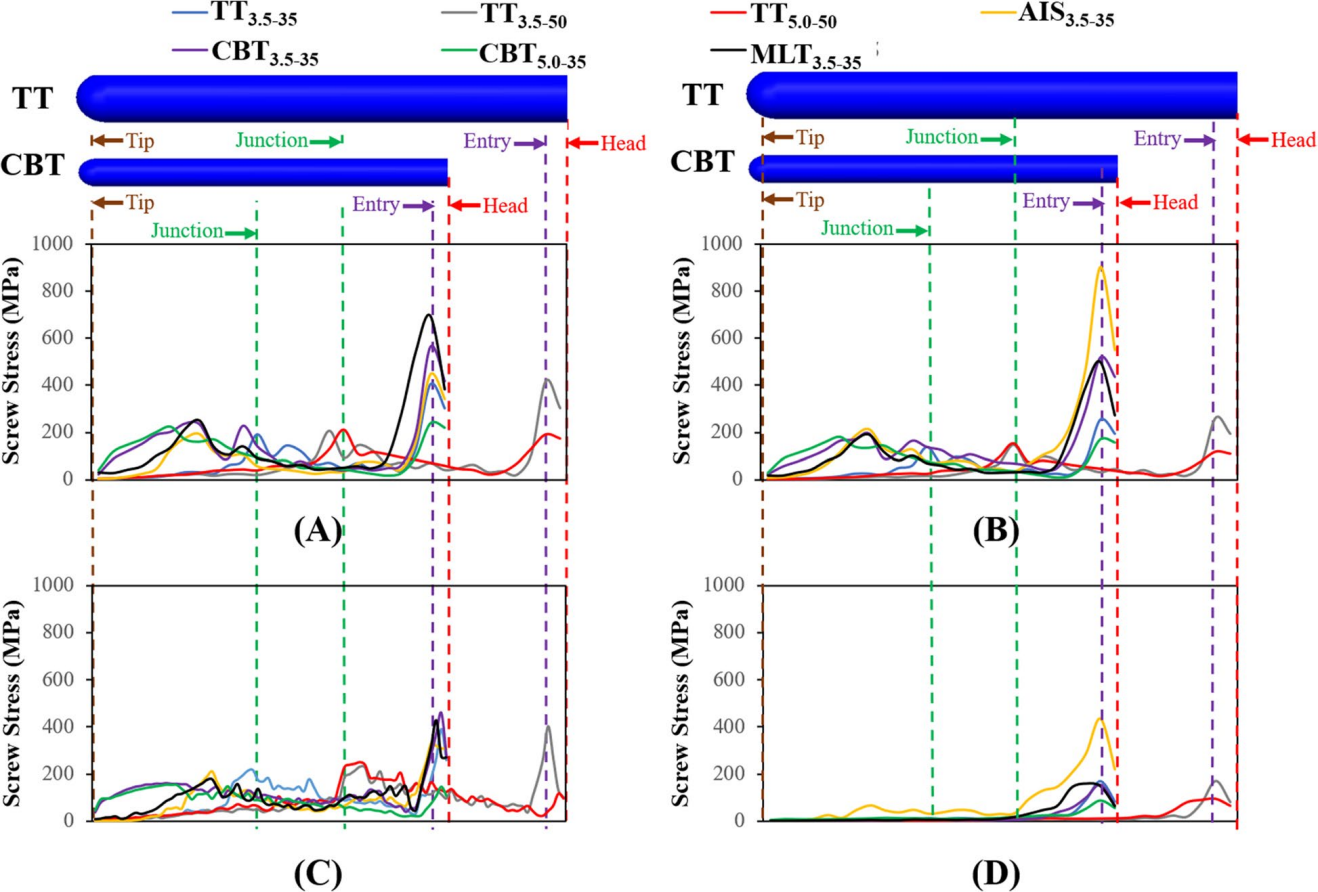
The distribution of screw stresses of the different fixators along the bone-screw interfaces (*Line AB*). **A** Flexion. **B** Extension. **C** Bending. **D** Rotation. The terms “Head”, “Entry”, “Junction”, and “Tip” denoted screw head (*i.e.,* screw hub), entry site of the screw into posterior element, interface between posterior element and cortical bone, and screw tip, respectively

Comparisons between the varieties are as follows: 1) trajectory: TT_3.5–35_ *vs.* CBT_3.5–35_ *vs.* AIS_3.5–35_ *vs.* MLT_3.5–35_, 2), diameter: TT_3.5–50_ *vs.* TT_5.0–50_ and CBT_3.5–35_ *vs.* CBT_5.0–35_ and 3) length: TT_3.5–35_ *vs.* TT_3.5–50_. Each group was designed to investigate how the screw parameters affected the tissue responses (Figs. 3, 4, 5 and 6) and implant behaviors (Fig. 7) for the fixed and adjacent segments.

For the fixed and adjacent discs, the stress-distributing contours of the healthy and instrumented models are shown in Fig. 3. Except bending, TT_3.5–35_, AIS_3.5–35_ and CBT_3.5–35_ of the trajectory group exhibited a stability compatible to the L4-L5 segment. Similar results were observed for CBT_5.0–3.5_ and MLT_5.0–35_. For bending, stabilization by TT was superior to its counterparts, although resulted in a more severe presentation of ASD (Fig. 3C). For rotation, kinematic problems associated with ASD were not observed.

Compared with TT_3.5–35_, the values for the normalized facet force at the L3-L4 segment decreased by an average of 45.9% for CBT_3.5–35_, across all motions (Figs. 4 and 5). For the TT_5.0–50_ and CBT_50-35_, decrease in facet force was 22.5%. For the disc response, decreases in ROM (stress) were: 24.8% (6.8%) for the TT_3.5–35_ and CBT_3.5–35_, respectively, and 8.5% (6.6%) for TT_5.0–50_ and CBT_5.0–3.5_, respectively.

No facet contact was simulated for flexion, extension for L4-L5 and rotation at the L4-L5 and L5-S1 segments (Fig. 6). The ASD progression at the L3-L4 segment was observed for all variations. Similar to the disc, the TT varieties resulted in a more severe presentation of ASD than their CBT counterparts. The increases in diameter and length strengthened the screw, leading to a higher facet force at the L3-L4 segment. For all varieties, ASDs of rotation were significantly less pronounced than those of the other motions. For bending, percentages related to the normalized facet force were negative across all varieties.

Along *Line AB*, two sites were marked as the boundary and material discontinuities: the screw-bone entry and the junction between the posterior element and vertebral body (Fig. 7). All screws were aligned at the tips for readers' clarity. Consistently, peak stresses for all varieties occurred near the screw hub. The slimmer screws experienced more stress in comparison to their counterparts, especially for extension. For all motions, the peak stress of the TT_3.5–50_ was on average 161.5% higher than that of TT_5.0–50_. Similarly, the difference in stress went up to 151.1% between the CBT_3.5–35_ and CBT_5.0–35_. Similar results were seen for longer screws. On average, peak stresses for TT_5.0–50_ were the lowest across the motions. For the trajectory effect, CBT_3.5–35_ was more stressed than TT_3.5–35_.

## Discussion

**Fig. 8:**
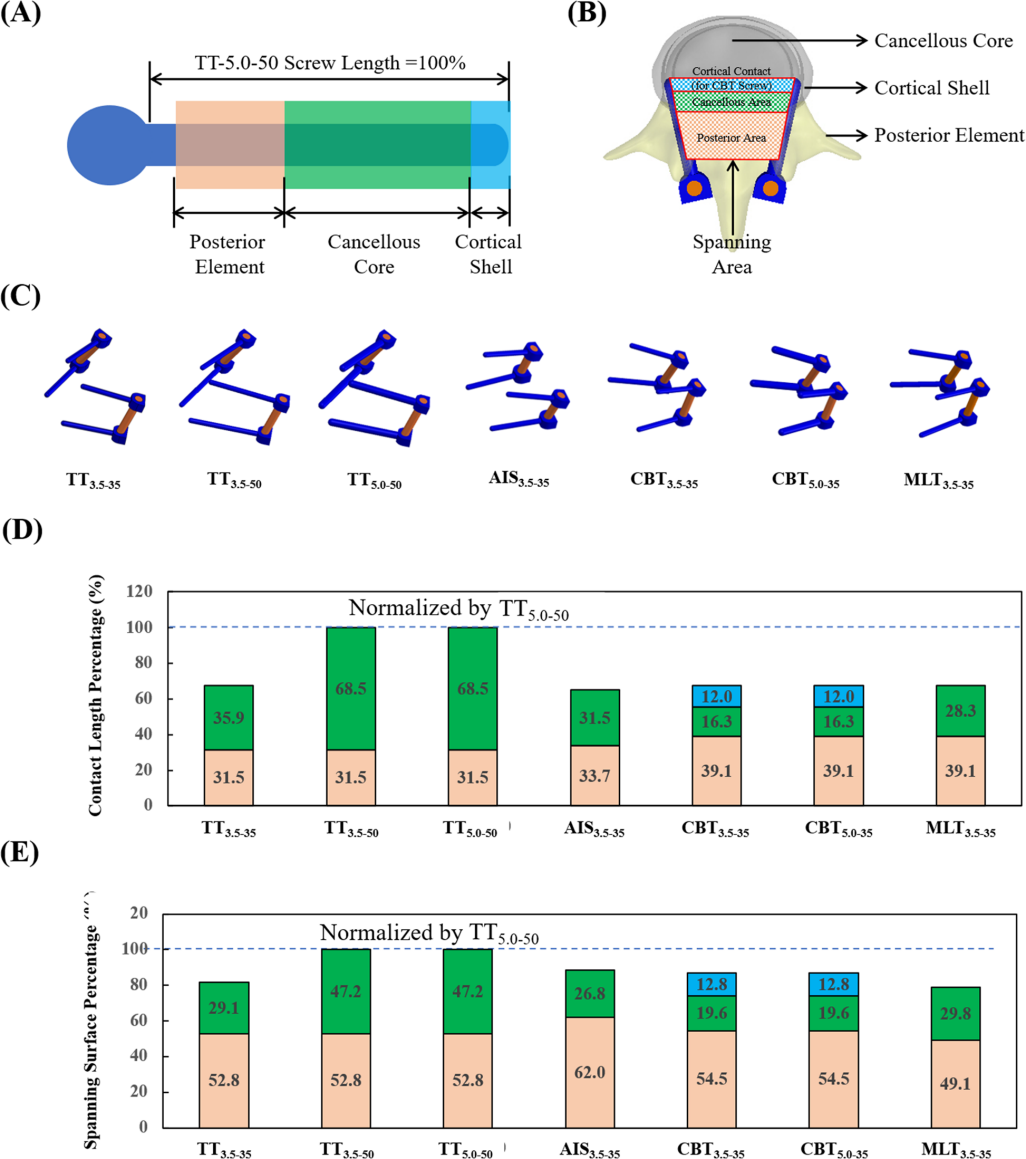
The schematic diagrams to illustrate the biomechanical effects of screw parameters on the bone-fixator construct. **A** The contact length within the different bones. **B** The spanning area of the paired screws within the different bones. **C** The screw-rod constructs were used to calculate the projected lengths and areas onto the transverse plane. **D** The normalized contact length percentage. **E** The normalized spanning area percentage. The length and surface percentages were normalized by the values of the TT_5.0–50_

The CBT aims to engage cortical bone within the pedicle and cortical shell at the screw tip. Along the screw length, there are three types of bone tissues: the posterior element, the cancellous bone and the cortical shell (Fig. 8A). In this study, the contact length percentages of the seven varieties within the three types of tissue were calculated and compared. The spanning area of the paired screws was defined as the area surrounded by the screw tips and hubs, and served as a base to anchor and stabilize the vertebral bone (Figs. 8B and C). Compared with the TT, the percentage of the normalized contact length of the CBT within the posterior element and cortical shell increased by 19.6% (Fig. 8D). Similarly, the percentage of the normalized spanning area of the CBT within the cortical bone increased by 14.5% (Fig. 8E). Collectively, this confirms the ability of the CBT screw to purchase the cortical bone [2, 21]. This may be one reason of reducing incidence of screw loosening because the osteoporotic process influence more on cancellous bone than on cortical bone [22]. Otherwise, the ideal trajectory of CBT screw is important to gain a reliable bone purchase [23]. Therefore, many studies provide 3D navigation or 3D printed guide for improving accuracy of CBT screw placement [15, 16].

All varieties provided stabilization at the fixed segment and led ASD compensation at the adjacent segment (Fig. 3). In general, the trajectory-induced effects exerted a stronger biomechanical impact on the adjacent facet joint than the disc (Figs. 4, 5 and 6). Compared with the TT_3.5–35_, the values of the normalized facet force at the L3-L4 segment decreased by an average of 45.9% for the CBT_3.5–35_, for all motions. For the TT_5.0–50_ and CBT_5.0–35_, the decrease in facet force was 22.5%. For the disc response, decreases in ROM (stress) were 24.8% (6.8%) for the TT_3.5–35_ and CBT_3.5–35_, respectively, and 8.5% (6.6%) for the TT_5.0–50_ and CBT_5.0–3.5_, respectively. These findings are consistent with clinical studies reporting that CBT can reduce ASD progression [5, 8, 10].

Ability to stabilize at the fixed segment was a chief aim in the fixation of the transpedicular screw. On average, the TT and CBT displayed a comparable ability to constrain the disc motion and share the disc load, except for bending and rotation (Figs. 4 and 5). It is possible that the divergent trajectory and shorter length of the CBT screws may have contributed to a reduced ability to stabilize the vertebral bone (Fig. 8C). The stronger CBT screw is recommended, given the increased screw stress and weakness in bending and rotation (Fig. 7). For flexion and rotation, the predicted ROM of the fixed disc among the 5.0 mm TT and CBT screws was consistent with the findings from Oshino et al. that the 4.5 mm CBT screws are comparable or even superior in ability to immobilize the inserted vertebra, compared to that of the 6.5 mm TT screws [7].

With a lateral- and upward- directed trajectory, the CBT screw was inevitably shorter and slimmer than the TT screw (Fig. 1B). Along the anatomical axis of the pedicle, however, the longer TT screw with a diameter between 5.5 and 6.0 mm could be employed. As such, the slimmer CBT screw may lead to potential fatigue breakage and even be insufficient to engage the cortical bone within the pedicle and cortical shell at the screw tip [11, 13, 14]. Compared with the TT_3.5–50_ and CBT_3.5–50_, the increased stresses of the TT_5.0–50_ and CBT_5.0–35_ indicate that the screw with the larger diameter should be used for larger-sized pedicles (Fig. 7) [13, 14]. When the diameter and length for the TT are the same, it consistently showed greater stress reductions than the CBT, especially for flexion and extension. Interestingly, this does not align with the finite element results reported by Matsukawa et al*.*, wherein they examined the effects on a vertebral bone with a simpler load [24, 25]. The nearly identical observed stresses on TT_3.5–35_ and TT_3.5–50_ reveals screw length exerts only a minor effect.

A few assumption-related limitations inherent to the finite element analysis have been described in finite-element models [17–19]. The screw threads and the bone-screw slippage were not simulated in this study, possibly leading to an overestimation the screws’ purchasing ability (especially regarding a greater contact by TT with the cancellous bone) and an underestimation of screw stresses (due to omitting the stress concentrations of the threads and cannulated holes). Unlike the micro-CT model from the study by Matsukawa et al*.* [24, 25], the microstructure of the trabecular bone was not modeled to simulate the distribution of the various types of bone tissue within the vertebral body. In addition, the morphological variations of the lumbar tissues were not systematically considered in this study. For some situations, there was no facet contact and significantly shortened CBT rods suppressed the ability of CBT to stabilize the L4-L5 segment in bending (Figs. 3C, 4C, and 6).

## Conclusions

In conclusion, the diameter and trajectory parameters had a stronger influence than length on the responses from the implant and tissues. The slimmer screws induced a significant amount of stress near the screw hub, especially for the CBT. For the CBT with the same values for diameter and length, it showed an equal or even superior stabilizing ability on the fixed segment compared to that of TT, though displayed a weaker progression of ASD. If the screw with the larger diameter can be used, the trajectory to recommend will depend on the type of surgery (*e.g.,* minimally invasive), the bone quality and the degree of degeneration at the adjacent segments. When the degeneration at the adjacent segments is considered, the CBT may provide the advantage of less stress at adjacent segments compared with TT. When the bone quality is considered, the CBT may provide more stiffer in osteoporotic segments than TT due to greater contact with the cortical bone and a wider supporting base between the paired screws; however, its screw placement (entry point and insertion trajectory) should be carefully executed in order to avoid breach of the vertebral body and ensure a stable bone-screw purchase.

## Data Availability

The datasets used and/or analyzed during the current study are available from the corresponding author on reasonable request.
